# Classroom participation among Chinese private college students: the role of teacher–student interaction, peer relationships, and learning motivation

**DOI:** 10.3389/fpsyg.2026.1673668

**Published:** 2026-04-15

**Authors:** Zhiyuan Yang, Connie Shin, Qijing Chen

**Affiliations:** 1 Faculty of International Education, Qingdao Hengxing University of Science and Technology, Qingdao, Shandong, China; 2 Faculty of Education and Sports Studies, Universiti Malaysia Sabah, Kota Kinabalu, Sabah, Malaysia

**Keywords:** classroom participation, direct and indirect impact, learning motivation, peer relationships, private college students, teacher–student interaction

## Abstract

**Introduction:**

Classroom participation is an important indicator of teaching effectiveness. However, limited research has simultaneously examined the roles of teacher–student interaction, peer relationships, and learning motivation in shaping classroom participation, particularly in private higher education contexts. This study aims to investigate the relationships among these variables among undergraduate students in a private university in Qingdao, China.

**Methods:**

A quantitative research design based on the positivist paradigm was adopted. Data were collected from a structured questionnaire from 534 undergraduate students. The data were analyzed using descriptive statistics, Pearson correlation analysis, and structural equation modeling (SEM).

**Results:**

The results indicate that classroom participation is positively associated with teacher–student interaction (*r* = 0.566, *p* < 0.01), peer relationships (r = 0.550, p < 0.01), and learning motivation (*r* = 0.618, *p* < 0.01). In addition, teacher–student interaction (*r* = 0.545, *p* < 0.01) and peer relationships (*r* = 0.544, *p* < 0.01) are positively associated with learning motivation. SEM results further show that learning motivation has the strongest effect on classroom participation (*β* = 0.364, *p* < 0.001), followed by teacher–student interaction (*β* = 0.252, *p* < 0.001)and peer relationships (*β* = 0.214, *p* < 0.001). Moreover, teacher–student interaction and peer relationships are indirectly associated with classroom participation through learning motivation.

**Conclusion:**

These findings highlight the interconnected roles of social interaction factors and learning motivation in understanding classroom participation. The study contributes to the literature by providing empirical evidence from private higher education and offers practical implications for improving teaching practices and educational policy.

## 1 Introduction

Classroom participation has long been recognized as a critical lens for assessing teaching effectiveness and institutional management (Fredricks et al., 2004). Its conceptual roots can be traced to the 1930s, when Tyler (1949) challenged traditional evaluation metrics by proposing ‘time invested in learning tasks’ as a proxy for student involvement. This shift laid the foundation for subsequent research linking student engagement to academic outcomes. In contemporary educational research, classroom participation is widely regarded not only as a behavioral indicator of student engagement but also as a key reflection of instructional quality. Educators have increasingly emphasized the role of active participation in enhancing learning effectiveness, and various pedagogical strategies have been proposed to promote student engagement and interaction in classroom settings (Goodman, 2016). Empirical studies have demonstrated that classroom participation is closely associated with students’ learning motivation and academic achievement (Akhtar et al., 2019). Students who actively participate in classroom activities tend to exhibit higher levels of motivation and show significant improvements in critical thinking and communication skills (Abubakar et al., 2017; Mohd Ali and Hassan, 2018). Furthermore, classroom participation provides an important perspective for understanding the dynamics of teacher–student interaction and the overall quality of the learning environment (Hofkens et al., 2023; Chang et al., 2022). Since the early 21st century, increasing attention has been devoted to this field, as researchers have recognized that classroom participation plays a significant predictive role in a wide range of students’ developmental and educational outcomes (Lam et al., 2014).

Although numerous studies have highlighted the significance of classroom participation (Fredricks et al., 2004; Da Luz, 2015; Robinson, 2022), most existing research has primarily focused on basic education rather than higher education contexts (Țepordei et al., 2023; Reeve and Lee, 2014). Moreover, despite the extensive body of literature examining factors associated with classroom participation, only a limited proportion of studies—approximately 23%—have been conducted within higher education settings (Cui, 2022). Scholars have therefore called for more research to develop a nuanced understanding of how social and motivational factors are related to classroom participation in higher education. In addition, prior studies often overlook contextual variables such as cultural and institutional differences, which may constrain the generalizability of their findings (Novitasari, 2020). From a theoretical perspective, this study is primarily grounded in Self-Determination Theory (SDT) (Ryan and Deci, 2020), which posits that students’ learning behaviors are driven by the fulfillment of three basic psychological needs: autonomy, competence, and relatedness. Within this framework, learning motivation is considered a central psychological mechanism associated with students’ engagement and participation behaviors. In higher education contexts, social interaction factors such as teacher–student interaction and peer relationships can be understood as critical environmental conditions related to the satisfaction of these psychological needs. Specifically, supportive teacher–student interaction may enhance students’ perceived competence and autonomy, while positive peer relationships contribute to a sense of relatedness. These environmental factors are therefore expected to be associated with classroom participation both directly and indirectly through learning motivation. Accordingly, this study adopts a social–motivational framework grounded in SDT, in which teacher–student interaction and peer relationships function as antecedent variables, learning motivation serves as a mediating mechanism, and classroom participation represents the behavioral outcome. Based on this theoretical framework, this study aims to examine the direct and indirect relationships among teacher–student interaction, peer relationships, learning motivation, and classroom participation within the context of Chinese private higher education. By integrating social interaction factors within a Self-Determination Theory framework, this study advances existing research by providing a context-specific explanation of classroom participation in Chinese private higher education, where student engagement dynamics differ from those in public institutions. The primary research question addressed in this study is:

What are the direct and indirect relationships among teacher–student interaction, peer relationships, learning motivation, and classroom participation?

## 2 Literature review

### 2.1 Theoretical framework

This study is theoretically grounded in Self-Determination Theory (SDT) (Ryan and Deci, 2020), which emphasizes the role of intrinsic and extrinsic motivation in shaping individuals’ behavior. SDT suggests that learning motivation arises when individuals’ basic psychological needs for autonomy, competence, and relatedness are satisfied. In educational contexts, classroom participation can be viewed as an observable behavioral manifestation of students’ motivation. Prior research has demonstrated that supportive social environments, particularly teacher–student interaction and peer relationships, play a critical role in fostering students’ psychological need satisfaction and motivation. Based on this theoretical perspective, this study conceptualizes teacher–student interaction and peer relationships as key environmental factors, learning motivation as the central psychological mechanism, and classroom participation as the outcome variable. This framework provides a coherent basis for hypothesis development and empirical testing.

### 2.2 Classroom participation

Classroom participation refers to students’ internal psychological engagement and observable learning behaviors during educational activities. It is widely regarded as a multidimensional construct encompassing behavioral, emotional, and cognitive components of engagement. Previous studies have shown that classroom participation is closely associated with academic performance, with both behavioral and emotional engagement serving as important predictors of learning outcomes (Bekkeringe and Ward, 2020; Aura et al., 2023). As an important indicator of educational quality, classroom participation has substantial practical relevance and provides valuable insights for improving teaching and learning environments (Tomaszewski et al., 2024). Prior research has also highlighted that structured instructional strategies are associated with more equitable classroom participation (Guenther and Abbott, 2024). Similarly, innovative teaching approaches, such as the flipped classroom model, have been found to be related to higher levels of student engagement across behavioral, emotional, and cognitive dimensions (Faro et al., 2024). In addition to its theoretical significance, classroom participation can be operationalized as a measurable construct in empirical research, enabling the assessment of students’ engagement in observable terms (Lopez, 2024). Accordingly, classroom participation is commonly used as a key indicator for understanding learning processes and evaluating educational effectiveness in higher education contexts (Snijders et al., 2020; Vänttinen, 2023).

### 2.3 Teacher–student interaction and classroom participation

The dynamics and characteristics of teacher–student interaction provide an important basis for understanding classroom participation (Ansari and Pianta, 2019). Supportive teacher–student interaction promotes classroom participation, while classroom-based emotional support correlates with students’ affective engagement and help-seeking behaviors (Pöysä et al., 2019; Liu et al., 2025). Teacher–student interaction not only influences engagement at the emotional and behavioral levels, but also plays an indirect role through learning motivation (Zee and Koomen, 2019; Burić et al., 2021; Radil et al., 2023). Longakit et al. (2025) examined the impact of teacher support on student classroom participation based on Self-Determination Theory. In highly engaging classrooms, teachers and students tend to demonstrate greater alignment in their perceptions of learning opportunities; however, in less engaging classrooms, students may fail to recognize the learning opportunities that teachers believe they are providing, suggesting potential communication gaps, mismatches in interests, or insufficient individual attention. Such mismatches may weaken learning motivation and reduce active engagement (Mercer and Dörnyei, 2020). Similarly, Ayuwanti et al. (2021) and Van Herpen et al. (2024) pointed out that teacher–student interaction can influence students’ autonomy, competence, and sense of belonging—the core elements of engagement described by Self-Determination Theory. However, both studies emphasize that, although teacher–student interaction significantly influences the development of these motivational factors, further research is needed to identify the specific types of interaction that most effectively enhance classroom participation. Building on these findings, the existing literature underscores the importance of integrating relational and motivational perspectives to better capture the complexity of classroom participation.

### 2.4 Peer relationships and classroom participation

Peer relationships play a significant role in the holistic development of students during childhood and adolescence. Previous studies have shown that harmonious peer relationships promote positive psychological development (Wang, 2024), and students with strong peer bonds are more likely to collaborate, exchange ideas, and support each other’s learning processes in the classroom (Qureshi et al., 2023). Empirical evidence further indicates that positive peer interactions contribute to a supportive classroom environment, enhance students’ sense of self-worth, and encourage active participation in classroom activities (Țepordei et al., 2023). In addition, students who are widely accepted by their classmates tend to demonstrate higher levels of classroom engagement (Wentzel et al., 2021; Knifsend et al., 2022). Mikami et al. (2017) also found that adolescents’ perceived peer relationships in the classroom consistently predict their self-reported behavioral engagement. From the perspective of Self-Determination Theory, peer relationships can be understood as a key social factor related to the fulfillment of students’ need for relatedness. Positive peer interactions may contribute to a sense of belonging and interpersonal connectedness, which are closely associated with the development of learning motivation. Through this motivational pathway, peer relationships may be linked to students’ classroom participation. Although much of the existing literature has focused on childhood and adolescence, these underlying social and motivational mechanisms are also relevant in higher education contexts. These findings suggest that peer relationships are associated with classroom participation among university students, both directly and indirectly through learning motivation.

### 2.5 Learning motivation and classroom participation

Learning motivation has been widely recognized as a central factor associated with students’ engagement and participation in educational settings. According to Self-Determination Theory (SDT), motivation can be broadly categorized into intrinsic and extrinsic forms, both of which are closely related to students’ learning behaviors (Ryan and Deci, 2020). In particular, intrinsic motivation—characterized by interest, enjoyment, and inherent satisfaction in learning activities—is consistently associated with higher levels of engagement and participation. Empirical studies have shown that learning motivation is positively associated with classroom participation and academic outcomes (Reeve, 2012). Students with higher levels of motivation tend to demonstrate greater involvement in classroom activities and more active learning behaviors. In addition, motivated students are more likely to engage in deeper cognitive processing, which is related to improved understanding of learning materials (Heilporn et al., 2024; Lei et al., 2024). Previous research has also indicated that learning motivation is closely related to social and instructional contexts. For example, classroom support and learning environments are associated with the development and maintenance of students’ motivation (Lazarides and Raufelder, 2017), and a reciprocal relationship has been observed between motivation and classroom participation (Bano and Riaz, 2023; Reeve and Lee, 2014). From the perspective of SDT, learning motivation can be conceptualized as a central psychological mechanism linking environmental factors and behavioral outcomes. Specifically, social interaction factors such as teacher–student interaction and peer relationships are related to students’ motivation through the satisfaction of basic psychological needs, which in turn is associated with classroom participation. Taken together, these findings suggest that learning motivation is closely associated with classroom participation and may function as a mediating mechanism in the relationship between social interaction factors and students’ engagement behaviors.

Based on Self-Determination Theory and prior empirical findings, the relationships among teacher–student interaction, peer relationships, learning motivation, and classroom participation can be theoretically derived. Specifically, supportive social interactions are expected to enhance students’ psychological need satisfaction, thereby increasing learning motivation, which in turn promotes classroom participation. Accordingly, the following hypotheses are proposed:

*H1:* Teacher–student interaction is positively associated with classroom participation.

*H2:* Peer relationships are positively associated with classroom participation.

*H3:* Learning motivation is positively associated with classroom participation.

*H4:* Teacher–student interaction is positively associated with learning motivation.

*H5:* Peer relationships are positively associated with learning motivation.

*H6:* Learning motivation mediates the relationship between teacher–student interaction and classroom participation.

*H7:* Learning motivation mediates the relationship between peer relationships and classroom participation.

## 3 Methodology

### 3.1 Research sample

This study targets undergraduate students in private universities in Shandong Province. Due to constraints in time, funding, and resources, it was not feasible to conduct a census of the entire target population. Therefore, this study focused on the accessible population, namely undergraduate students at Qingdao Hengxing College. According to the 2023 Provincial Teaching Quality Report issued by the Shandong Provincial Department of Education, the total number of undergraduate students at the institution was 17,060 (Department of Education of Shandong Province, 2024). A stratified random sampling method was employed. The sample was first divided into strata based on students’ grade level (first-year to fourth-year) and gender. Random sampling was then conducted within each stratum. Considering the requirements of multivariate statistical analysis, particularly structural equation modeling (SEM), and to ensure sufficient cases for subgroup-level analysis, each stratum was required to include no fewer than 50 respondents (Hair et al., 2019). This approach helps improve the stability of parameter estimates and reduces potential sampling bias. Rather than strictly adhering to proportional allocation, the sampling strategy aimed to balance subgroup representation and analytical feasibility. A total of 580 questionnaires were distributed, and 534 valid responses were obtained, yielding an effective response rate of 92%. The final sample size was adequate for subsequent statistical analyses, including structural equation modeling. Although efforts were made to enhance representativeness through stratified sampling, the sample was drawn from a single private university in Qingdao, Shandong Province. Therefore, the findings should be interpreted with caution when generalizing to other institutional or regional contexts. However, while the sample is drawn from a single institution, it reflects typical characteristics of Chinese private higher education.

### 3.2 Research instruments

This study employed a structured questionnaire to collect data from undergraduate students. All measurement instruments were adapted from well-established and validated scales in previous studies. To ensure their suitability for the context of private higher education in China, all instruments originally developed in English were translated into Chinese using a standard translation and back-translation procedure to ensure semantic equivalence, with minor contextual modifications made to align the items with the higher education setting. To ensure content validity, the questionnaire was reviewed by two professors specializing in educational management. In addition, a pilot study was conducted with 71 undergraduate students, during which items with ambiguous wording, redundancy, or weak relevance to the research objectives were removed. As a result, a total of 81 items were retained for the final survey.

All constructs were measured using a five-point Likert scale ranging from 1 (strongly disagree) to 5 (strongly agree). Classroom participation, as the dependent variable, was measured using a 17-item scale adapted from Handelsman et al. (2005), covering four dimensions: skills participation, emotional participation, interaction participation, and performance participation, with Cronbach’s alpha values ranging from 0.846 to 0.903 for this scale. Teacher–student interaction was measured using a 23-item scale adapted from Fisher et al. (1995), including leadership, helping/friendly behavior, understanding, responsibility/freedom, and strictness, with Cronbach’s alpha values ranging from 0.811 to 0.904. Peer relationships were assessed using a 21-item scale adapted from Aydoğdu (2022), covering intimacy, popularity, trust, and insightfulness, with Cronbach’s alpha values ranging from 0.809 to 0.925. Although this scale was originally developed for adolescents, the construct remains relevant in undergraduate contexts. Learning motivation was measured using a 20-item scale adapted from McKeachie et al. (1986), including goal orientation, task value, and self-efficacy for learning and performance, with reported Cronbach’s alpha values ranging from 0.916 to 0.963. Overall, all measurement instruments demonstrated satisfactory reliability and were appropriate for subsequent statistical analysis.

### 3.3 Data analysis

To assess the potential presence of common method bias (CMB), both procedural and statistical remedies were applied. Procedurally, the questionnaire was administered anonymously and participation was voluntary, which helps reduce evaluation apprehension and response bias. Statistically, Harman’s single-factor test was conducted by subjecting all measurement items to an unrotated exploratory factor analysis. The results indicated that multiple factors with eigenvalues greater than 1 were extracted, and the first factor accounted for 31.23% of the total variance, which is below the commonly adopted threshold of 40% (Podsakoff et al., 2003). Therefore, common method bias is unlikely to be a serious concern in this study.

Subsequently, the data were analyzed using SPSS 29.0 and AMOS 27.0. Descriptive statistics, including means, standard deviations, skewness, and kurtosis, were calculated to examine the distribution characteristics of the variables. Pearson correlation analysis was conducted to assess the relationships among the variables. Furthermore, structural equation modeling (SEM) was employed to test the hypothesized relationships among the constructs. Model fit indices were evaluated to determine the adequacy of the proposed model and the validity of the theoretical framework.

## 4 Results

Table 1 presents the demographic characteristics of the sample, including the distribution of gender and grade levels. Among the 534 respondents, 275 were female (51.5%) and 259 were male (48.5%). In terms of grade, 101 students were in their first year (18.9%), 157 in their second year (29.4%), 158 in their third year (29.6%), and 118 in their fourth year (22.1%). Although the sample distribution does not perfectly match the overall population structure, it demonstrates a relatively balanced composition across gender and grade levels, which provides an adequate basis for subsequent statistical analyses.

**Table 1:** Demographic characteristics.

Demographic variables	Categories	Frequency	Percentage (%)
Gender	Male	259	48.5
	Female	275	51.5
Grade	Grade 1	101	18.9
	Grade 2	157	29.4
	Grade 3	158	29.6
	Grade 4	118	22.1

Table 2 presents the descriptive statistics for all study variables, including means, standard deviations, skewness, and kurtosis. The mean scores of the four variables were at a moderate-to-upper level: teacher–student interaction (*M* = 3.13, *SD* = 0.92), peer relationships (*M* = 3.28, *SD* = 0.83), learning motivation (*M* = 3.22, *SD* = 0.88), and classroom participation (*M* = 3.07, *SD* = 0.94). Among these variables, peer relationships had the highest mean score, while classroom participation had the lowest. The skewness values ranged from −0.093 to 0.055, and the kurtosis values ranged from −1.336 to −1.125. All skewness and kurtosis values fell within the commonly accepted range of −2 to +2, indicating that the data approximate a normal distribution.

**Table 2:** Mean, standard, skewness, and kurtosis scores of variables.

Variable	*N*	Mean	Std. Deviation	Skewness	Kurtosis		
				Statistic	Std. Error	Statistic	Std. Error
TS	534	3.1324	0.92245	−0.093	0.106	−1.246	0.211
PR	534	3.2871	0.83571	0.024	0.106	−1.125	0.211
LM	534	3.2239	0.88221	−0.01	0.106	−1.191	0.211
CE	534	3.0788	0.94514	0.055	0.106	−1.336	0.211

TS, teacher–student interaction; PR, peer relationships; LM, learning motivation; CE, classroom participation.

Table 3 presents the reliability analysis results for all study variables. Cronbach’s *α* and composite reliability (CR) were used to assess the internal consistency of each construct. The results indicate that teacher–student interaction (α = 0.951, C*r* = 0.962), peer relationships (α = 0.929, C*r* = 0.941), learning motivation (α = 0.937, C*r* = 0.950), and classroom participation (α = 0.936, C*r* = 0.966) all demonstrated high levels of reliability. All Cronbach’s α and CR values exceeded the recommended threshold of 0.70, indicating satisfactory internal consistency and supporting the use of these constructs for subsequent analyses.

**Table 3:** Reliability analysis of the study variables.

Construct	Cronbach’s alpha	Composite reliability
TS	0.951	0.962
PR	0.929	0.941
LM	0.937	0.950
CE	0.936	0.966

TS, teacher–student interaction; PR, peer relationships; LM, learning motivation; CE, classroom participation.

Table 4 presents the validity analysis of all study variables. Construct validity for each latent variable was assessed using factor loadings and the Average Variance Extracted (AVE). The factor loadings for teacher–student interaction ranged from 0.784 to 0.885, with an AVE of 0.721. Peer relationships had factor loadings between 0.697 and 0.875, and an AVE of 0.711. Learning motivation showed factor loadings ranging from 0.829 to 0.879, with an AVE of 0.742. classroom participation had factor loadings between 0.764 and 0.952, and an AVE of 0.753. All measurement items demonstrated factor loadings above the recommended threshold of 0.50, and all constructs had AVE values exceeding the minimum standard of 0.50 (Hair et al., 2019), indicating that all latent variables in this study exhibit good convergent validity and are suitable for subsequent structural modeling analyses.

**Table 4:** Validity analysis of the study variables.

Construct	Factor loadings	Average variance extracted (AVE)
TS	0.784–0.885	0.721
PR	0.697–0.875	0.711
LM	0.829–0.879	0.742
CE	0.764–0.952	0.753

TS, teacher–student interaction; PR, peer relationships; LM, learning motivation; CE, classroom participation.

Table 5 presents the correlation analysis results for all study variables. Teacher–student interaction was positively correlated with classroom participation (*r* = 0.566, *p* < 0.01). Peer relationships were also positively correlated with classroom participation (*r* = 0.550, *p* < 0.01). Learning motivation showed the strongest correlation with classroom participation (*r* = 0.618, *p* < 0.01). In addition, significant positive correlations were observed among the independent variables. Teacher–student interaction (*r* = 0.545, *p* < 0.01) and peer relationships (*r* = 0.544, *p* < 0.01) were both moderately correlated with learning motivation. Overall, these results indicate that the key variables are significantly associated with one another, providing preliminary support for the hypothesized relationships and justifying the use of structural equation modeling for further analysis.

**Table 5:** Relationship between teacher–student interaction, peer relationships, learning motivation and classroom participation.

Variables	Statistics	TS	PR	LM	CE
TS	Pearson correlation	1	0.542	0.545	0.566
	Sig. (2-tailed)		<0.001	<0.001	<0.001
PR	Pearson correlation	0.542	1	0.544	0.550
	Sig. (2-tailed)	<0.001		<0.001	<0.001
LM	Pearson correlation	0.545	0.544	1	0.618
	Sig. (2-tailed)	<0.001	<0.001		<0.001
CE	Pearson correlation	0.566	0.550	0.618	1
	Sig. (2-tailed)	<0.001	<0.001	<0.001	

TS, teacher–student interaction; PR, peer relationships; LM, learning motivation; CE, classroom participation.

Table 6 presents the model fit indices of the structural equation model (SEM). The results indicate that the model demonstrates an acceptable fit to the data. Specifically, the RMSEA value is 0.033, which is below the recommended threshold of 0.05. The comparative fit indices are also within acceptable ranges, with CFI = 0.998, TLI = 0.996, and IFI = 0.998, all exceeding the suggested cutoff of 0.95. In addition, the CMIN/DF value is 1.579, which falls within the acceptable range. Overall, these fit indices suggest that the structural model provides a good fit to the data.

**Table 6:** Result of assessing fit model.

Type of fit	Fit measure	Index	Interpretation
Absolute fit	RMSEA	0.033	Good Fit
Incremental fit	CFI	0.998	Good Fit
	TLI	0.996	Good Fit
	IFI	0.998	Good Fit
Chi-square	CMIN/DF	1.579	Acceptable fit

**Figure 1:**
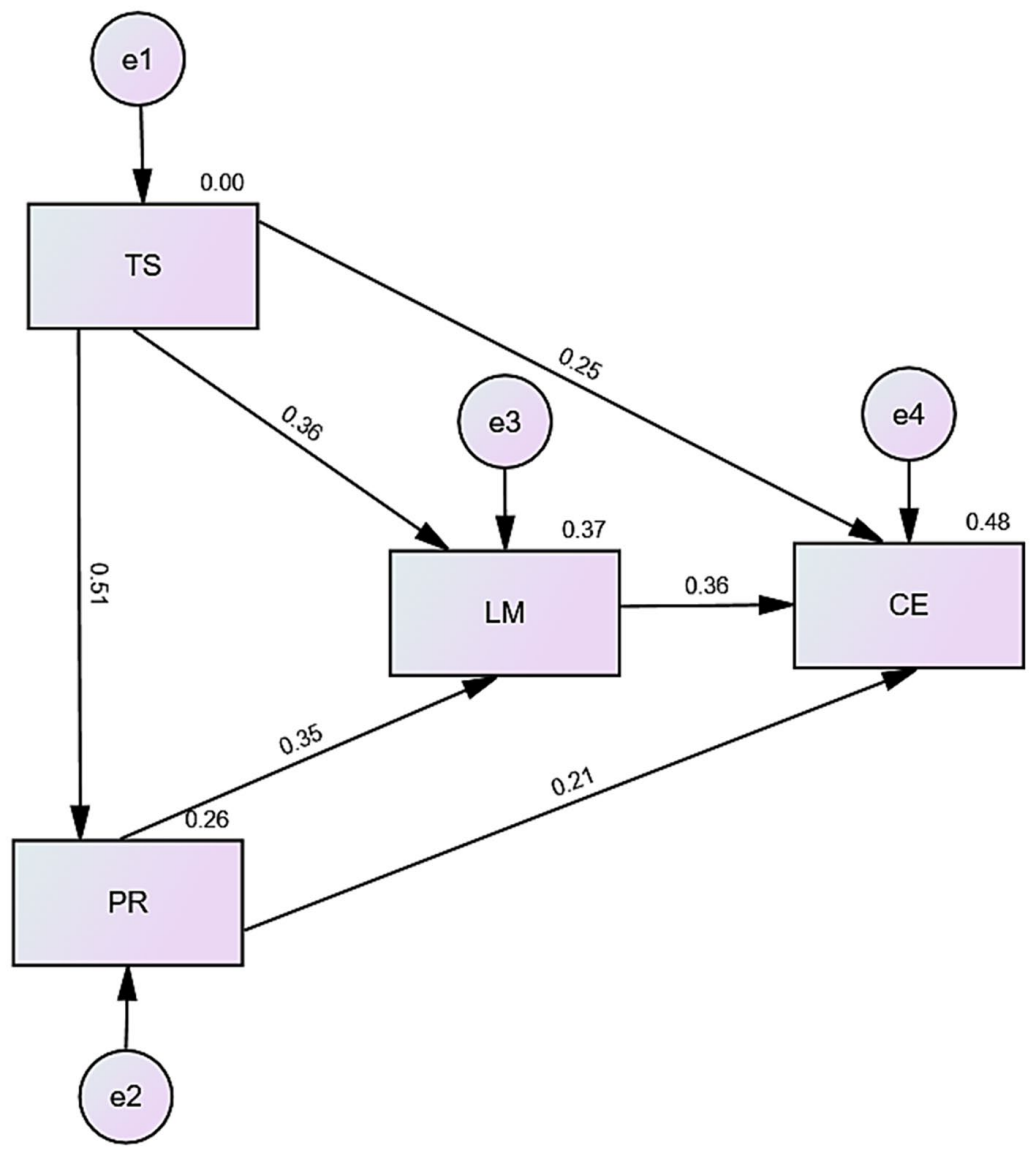
Structural equation model (SEM) of the relationships among teacher–student interaction, peer relationships, learning motivation and classroom participation TS: teacher–student interaction, PR: peer relationships, LM: learning motivation, CE: classroom participation.

Table 7 and Figure 1 present the direct relationships among teacher–student interaction, peer relationships, learning motivation, and classroom participation among undergraduate students in private colleges. The findings indicate that learning motivation shows the strongest association with classroom participation (*β* = 0.364, *p* < 0.001), followed by teacher–student interaction (*β* = 0.252, *p* < 0.001) and peer relationships (*β* = 0.214, *p* < 0.001). The results also indicate that teacher–student interaction is positively associated with learning motivation (*β* = 0.357, *p* < 0.001). Similarly, peer relationships are positively associated with learning motivation (*β* = 0.347, *p* < 0.001). All reported associations are statistically significant.

**Table 7:** Direct effects of structural equation model (SEM).

Path	Standardized path coefficient (β)	Lower bound (95% CI)	Upper bound (95% CI)	*p*-value
TS → CE	0.252	0.159	0.348	<0.001
PR → CE	0.214	0.128	0.296	<0.001
LM → CE	0.364	0.280	0.445	<0.001
TS → LM	0.357	0.279	0.438	<0.001
PR → LM	0.347	0.260	0.432	<0.001

TS, teacher–student interaction; PR, peer relationships; LM, learning motivation; CE, classroom participation.

Table 8 presents the indirect effects of teacher–student interaction and peer relationships on classroom participation through learning motivation. The mediation effects were tested using 5,000 bootstrap resamples and 95% bias-corrected confidence intervals. Consistent with Hypothesis H6, teacher–student interaction exhibited a significant indirect effect on classroom participation via learning motivation (*β* = 0.129, *p* < 0.01), with a 95% bias-corrected bootstrap confidence interval of [0.094, 0.177]. In support of Hypothesis H7, peer relationships also demonstrated a significant indirect effect on classroom participation through learning motivation (*β* = 0.128, *p* < 0.01), with a 95% bias-corrected bootstrap confidence interval of [0.087, 0.178]. These findings provide evidence that learning motivation functions as a mediating variable in the relationships between social interaction factors and classroom participation.

**Table 8:** Indirect effects of structural equation model (SEM).

Path	Standardized indirect effect (β)	Lower bound (95% CI)	Upper bound (95% CI)	*p-*value
TS → CE	0.129	0.094	0.177	0.001
PR → CE	0.128	0.087	0.178	<0.001

TS, teacher–student interaction; PR, peer relationships; LM, learning motivation; CE, classroom participation.

## 5 Discussion

Guided by Self-Determination Theory (SDT), the findings of this study provide empirical support for a social–motivational mechanism in which social interaction factors influence classroom participation both directly and indirectly through learning motivation. Specifically, teacher–student interaction and peer relationships function as key environmental conditions, while learning motivation operates as a central psychological mechanism linking these factors to students’ behavioral engagement.

First, the findings indicate that learning motivation shows the strongest association with classroom participation. This result is consistent with SDT (Ryan and Deci, 2020), which posits that students with higher levels of intrinsic motivation are more likely to engage actively and persist in learning activities. Importantly, this relationship appears particularly salient in the context of Chinese private higher education, where students often exhibit heterogeneous academic backgrounds and varying levels of learning preparedness. In such contexts, students may rely more heavily on internal motivational resources to sustain engagement, making learning motivation a critical driver of classroom participation.

Second, teacher–student interaction is positively associated with classroom participation. This finding aligns with prior research emphasizing the importance of supportive instructional environments (Ayuwanti et al., 2021; Longakit et al., 2025). From the perspective of SDT, teacher–student interaction may enhance students’ perceived competence and autonomy by providing guidance, feedback, and encouragement, thereby strengthening their willingness to participate. In private higher education settings, where instructional resources and teaching support may be relatively limited, effective teacher–student interaction becomes particularly important in fostering a supportive learning climate and promoting student engagement.

Third, peer relationships are also positively associated with classroom participation. Students who experience supportive peer interactions are more likely to develop a sense of belonging and psychological safety, which facilitates active engagement in classroom activities. From an SDT perspective, peer relationships primarily satisfy students’ need for relatedness, which plays a crucial role in sustaining motivation and participation. In addition, in contexts where teacher support may be constrained, peer interaction can serve as an important supplementary source of academic and emotional support.

Furthermore, the results reveal that teacher–student interaction and peer relationships influence classroom participation indirectly through learning motivation, highlighting the importance of an underlying psychological mechanism. Rather than affecting participation solely at the behavioral level, these social interaction factors operate by shaping students’ internal motivational states. From the perspective of Self-Determination Theory, supportive teacher and peer environments contribute to the satisfaction of students’ needs for autonomy, competence, and relatedness, which in turn enhances their intrinsic motivation. This increased motivation then translates into higher levels of active classroom participation. This finding underscores that student engagement is not merely a direct response to external conditions, but is fundamentally driven by internalized motivational processes.

Unlike prior studies that have primarily examined isolated or direct relationships among teacher–student interaction, peer relationships, and learning motivation (Ayuwanti et al., 2021; Van Herpen et al., 2024; Longakit et al., 2025), this study demonstrates an integrated pathway in which social interaction factors jointly influence classroom participation through a mediating motivational mechanism. By situating these relationships within a unified SDT-based framework, the present study provides a more comprehensive explanation of classroom participation, particularly in the context of Chinese private higher education.

These findings are particularly meaningful for private higher education institutions in China, where instructional resources are often limited, teaching workloads are relatively high, and students’ academic foundations may vary considerably. In such contexts, social interaction may function as a compensatory mechanism, while learning motivation serves as a critical driver of engagement. Enhancing classroom participation therefore requires not only instructional adjustments but also systematic efforts to strengthen both social interaction and student motivation.

Based on these findings, several practical implications can be derived. Consistent with the SDT framework, effective teaching strategies should aim to enhance students’ autonomy, competence, and relatedness. First, teachers are encouraged to adopt structured interaction strategies, such as think–pair–share, guided questioning, and small-group discussions, to increase opportunities for participation without substantially increasing instructional workload. Second, given the diversity in students’ academic preparedness, differentiated instructional approaches—such as tiered tasks and flexible participation formats—can support students at different levels in gradually engaging with classroom activities. Third, fostering positive peer relationships is essential; collaborative learning activities, peer feedback tasks, and group-based assignments can strengthen peer interaction and create a supportive classroom climate. Finally, enhancing students’ learning motivation should remain a central focus. Providing autonomy-supportive learning environments, incorporating real-world relevance, and offering timely positive feedback can help strengthen intrinsic motivation and sustain long-term engagement in classroom learning.

## 6 Conclusion

This study examined the relationships among teacher–student interaction, peer relationships, learning motivation, and classroom participation in the context of Chinese private higher education. The findings indicate that learning motivation shows the strongest association with classroom participation, followed by teacher–student interaction and peer relationships. In addition, teacher–student interaction and peer relationships are indirectly associated with classroom participation through learning motivation, highlighting the role of motivational processes in linking social interaction factors with student engagement.

These findings provide empirical support for the social-motivational framework of classroom participation and contribute to the literature by examining the integrated relationships among social interaction, learning motivation, and classroom participation within a unified analytical model.

From a practical perspective, the results highlight the importance of enhancing both social interaction and learning motivation to improve classroom participation. Teachers and institutions should adopt appropriate instructional and collaborative approaches to create supportive and engaging learning environments.

Despite these contributions, several limitations should be acknowledged. First, the sample was drawn from a single private university, which may limit the generalizability of the findings. Second, although statistical tests suggest that common method bias is not a serious concern, all variables were measured using self-report data, which may still introduce potential bias. Third, due to the cross-sectional design, causal relationships cannot be established, and the findings reflect associations rather than causal effects.

Future research may extend the sample to include public universities and vocational institutions, and employ multiple data sources such as classroom observations, interviews, and behavioral data to enhance the robustness of the findings. Further studies may also explore additional contextual and institutional factors to better understand the complexity of classroom participation in diverse educational settings.

## Data Availability

The raw data supporting the conclusions of this article will be made available by the authors, without undue reservation.
