# Correction: Classroom participation among Chinese private college students: the role of teacher–student interaction, peer relationships, and learning motivation

**DOI:** 10.3389/fpsyg.2026.1866512

**Published:** 2026-05-07

**Authors:** Frontiers Production Office

**Affiliations:** Frontiers Media SA, Lausanne, Switzerland

**Keywords:** classroom participation, direct and indirect impact, learning motivation, peer relationships, private college students, teacher–student interaction

In the abstract, an incorrect version of the **Conclusion** section was published. This has been corrected to read:

**Conclusion:** These findings highlight the interconnected roles of social interaction factors and learning motivation in understanding classroom participation. The study contributes to the literature by providing empirical evidence from private higher education and offers practical implications for improving teaching practices and educational policy.

The original version of this article has been updated.

